# Health insurance coverage with or without a nurse-led task shifting strategy for hypertension control: A pragmatic cluster randomized trial in Ghana

**DOI:** 10.1371/journal.pmed.1002561

**Published:** 2018-05-01

**Authors:** Gbenga Ogedegbe, Jacob Plange-Rhule, Joyce Gyamfi, William Chaplin, Michael Ntim, Kingsley Apusiga, Juliet Iwelunmor, Kwasi Yeboah Awudzi, Kofi Nana Quakyi, Jazmin Mogaverro, Kiran Khurshid, Bamidele Tayo, Richard Cooper

**Affiliations:** 1 Department of Population Health, New York University School of Medicine, New York, New York, United States of America; 2 School of Medical Sciences, Kwame Nkrumah University of Science and Technology, Kumasi, Ghana; 3 Department of Psychology, St. John’s University, Queens, New York, United States of America; 4 College for Public Health and Social Justice, Saint Louis University, St. Louis, Missouri, United States of America; 5 Ashanti Regional Health Directorate, Ghana Health Service, Ashanti, Ghana; 6 College of Global Public Health, New York Unversity, New York, New York, United States of America; 7 Department of Public Health Sciences, Stritch School of Medicine, Loyola University Medical Center, Maywood, Illinois, United States of America; University of Oxford, UNITED KINGDOM

## Abstract

**Background:**

Poor access to care and physician shortage are major barriers to hypertension control in sub-Saharan Africa. Implementation of evidence-based systems-level strategies targeted at these barriers are lacking. We conducted a study to evaluate the comparative effectiveness of provision of health insurance coverage (HIC) alone versus a nurse-led task shifting strategy for hypertension control (TASSH) plus HIC on systolic blood pressure (SBP) reduction among patients with uncontrolled hypertension in Ghana.

**Methods and findings:**

Using a pragmatic cluster randomized trial, 32 community health centers within Ghana’s public healthcare system were randomly assigned to either HIC alone or TASSH + HIC. A total of 757 patients with uncontrolled hypertension were recruited between November 28, 2012, and June 11, 2014, and followed up to October 7, 2016. Both intervention groups received health insurance coverage plus scheduled nurse visits, while TASSH + HIC comprised cardiovascular risk assessment, lifestyle counseling, and initiation/titration of antihypertensive medications for 12 months, delivered by trained nurses within the healthcare system. The primary outcome was change in SBP from baseline to 12 months. Secondary outcomes included lifestyle behaviors and blood pressure control at 12 months and sustainability of SBP reduction at 24 months. Of the 757 patients (389 in the HIC group and 368 in the TASSH + HIC group), 85% had 12-month data available (60% women, mean BP 155.9/89.6 mm Hg). In intention-to-treat analyses adjusted for clustering, the TASSH + HIC group had a greater SBP reduction (−20.4 mm Hg; 95% CI −25.2 to −15.6) than the HIC group (−16.8 mm Hg; 95% CI −19.2 to −15.6), with a statistically significant between-group difference of −3.6 mm Hg (95% CI −6.1 to −0.5; *p* = 0.021). Blood pressure control improved significantly in both groups (55.2%, 95% CI 50.0% to 60.3%, for the TASSH + HIC group versus 49.9%, 95% CI 44.9% to 54.9%, for the HIC group), with a non-significant between-group difference of 5.2% (95% CI −1.8% to 12.4%; *p* = 0.29). Lifestyle behaviors did not change appreciably in either group. Twenty-one adverse events were reported (9 and 12 in the TASSH + HIC and HIC groups, respectively). The main study limitation is the lack of cost-effectiveness analysis to determine the additional costs and benefits, if any, of the TASSH + HIC group.

**Conclusions:**

Provision of health insurance coverage plus a nurse-led task shifting strategy was associated with a greater reduction in SBP than provision of health insurance coverage alone, among patients with uncontrolled hypertension in Ghana. Future scale-up of these systems-level strategies for hypertension control in sub-Saharan Africa requires a cost–benefit analysis.

**Trial registration:**

ClinicalTrials.gov NCT01802372

## Abstract 2

In a cluster randomized trial, Gbenga Ogedegbe and colleageues examine the effectiveness of a nurse-led task shifting strategy for hypertension control in Ghana

## Author summary

**Why was this study done?:**

- The burden of cardiovascular diseases in sub-Saharan Africa (SSA) is growing due to an increased prevalence of uncontrolled hypertension.
- Poor access to healthcare and physician shortage are major barriers to hypertension control in SSA.
- Implementation of evidence-based systems-level strategies targeted at these barriers are lacking.

**What did the researchers do and find?:**

- In this cluster randomized controlled trial conducted among 757 patients across 32 community health centers in Ghana, we examined whether the addition of a nurse-led intervention for hypertension control to health insurance coverage led to a greater reduction in systolic blood pressure (BP) than provision of health insurance coverage alone.
- We found that the addition of a nurse-led intervention to provision of health insurance coverage led to a greater reduction in systolic BP (−20.4 mm Hg) than health insurance coverage alone (−16.8 mm Hg), with a net difference in reduction of 3.6 mm Hg (*p* = 0.021) at 12 months.

**What do these findings mean?:**

- The study findings provide evidence for policy makers to recommend addition of a nurse-led task shifting strategy for hypertension control to health insurance coverage for management of hypertension in SSA.
- Future scale-up of this nurse-led strategy in SSA would require a cost–benefit analysis and establishment of a policy that grants nurses prescribing power to treat patients with uncomplicated hypertension, similar to current policy for treatment of HIV.

## Introduction

Ghana and other countries in sub-Saharan Africa (SSA) are experiencing a growing burden of cardiovascular diseases (CVDs) propelled by a rapidly increasing prevalence of hypertension [1]. Barriers to hypertension control in SSA include poor access to healthcare due to lack of health insurance coverage, high out-of-pocket costs, and shortage of skilled healthcare providers [2,3]. The World Health Organization (WHO) estimates that 60% of countries worldwide fall below the threshold of what is considered a sufficient level of skilled health professionals, i.e., they have fewer than 59.4 physicians, nurses, or midwives per 10,000 population [4]. Most countries in SSA fall far below this threshold, with a per capita skilled workforce of less than 22.8 per 10,000. Specifically, there are only 2 physicians and 11 nurses per 10,000 people in SSA, compared to 19 physicians and 49 nurses per 10,000 in North America [4]. The healthcare workforce crisis is even more acute in Ghana, which in 2015 had 1 physician and 9 nurses per 10,000 people [4], thus limiting its capacity for hypertension control at the primary care level, where most people receive their care.

Given such limited resources, systems-level strategies such as task shifting and provision of health insurance coverage are needed to mitigate the growing burden of hypertension-related morbidity in SSA. Task shifting, which is defined as the rational distribution of tasks among the healthcare workforce, is especially useful in low- and middle-income countries (LMICs) facing healthcare human resources crises [5], given its benefits in the management of HIV/AIDS [6]. Task shifting of primary care duties from physicians to nurses or non-physician workers is a low-cost strategy for mitigating systems-level barriers to optimal hypertension control in LMICs because it utilizes multiple strategies for management of CVD including screening, counseling on lifestyle modification, initiation of treatment, and referral to specialist care [5,7,8]. Although in 2010 WHO evaluated the clinical effectiveness of the WHO CVD-Risk Management Package (WHO CVD Package) for management of hypertension by non-physician healthcare workers in Nigeria and China [9], its implementation and sustainability in SSA remains untested, particularly compared to other systems-level strategies like provision of health insurance coverage within an existing healthcare system. Ghana, with its established national health insurance scheme, provides an ideal platform to evaluate the implementation and sustainability of the WHO CVD Package for hypertension control.

Using a comparative effectiveness research strategy, we compared the effectiveness of 2 systems-level strategies, provision of health insurance coverage (HIC) alone versus a nurse-led task shifting strategy for hypertension control (TASSH) plus HIC, on systolic blood pressure (SBP) reduction among patients with newly diagnosed uncontrolled hypertension in 32 community health centers (CHCs) in Ghana. TASSH comprised implementation of the WHO CVD Package (cardiovascular [CV] risk assessment, patient counseling on lifestyle modification, and initiation and titration of antihypertensive medications), delivered by trained community health nurses [10].

## Methods

### Study design and participants

**Fig 1:**
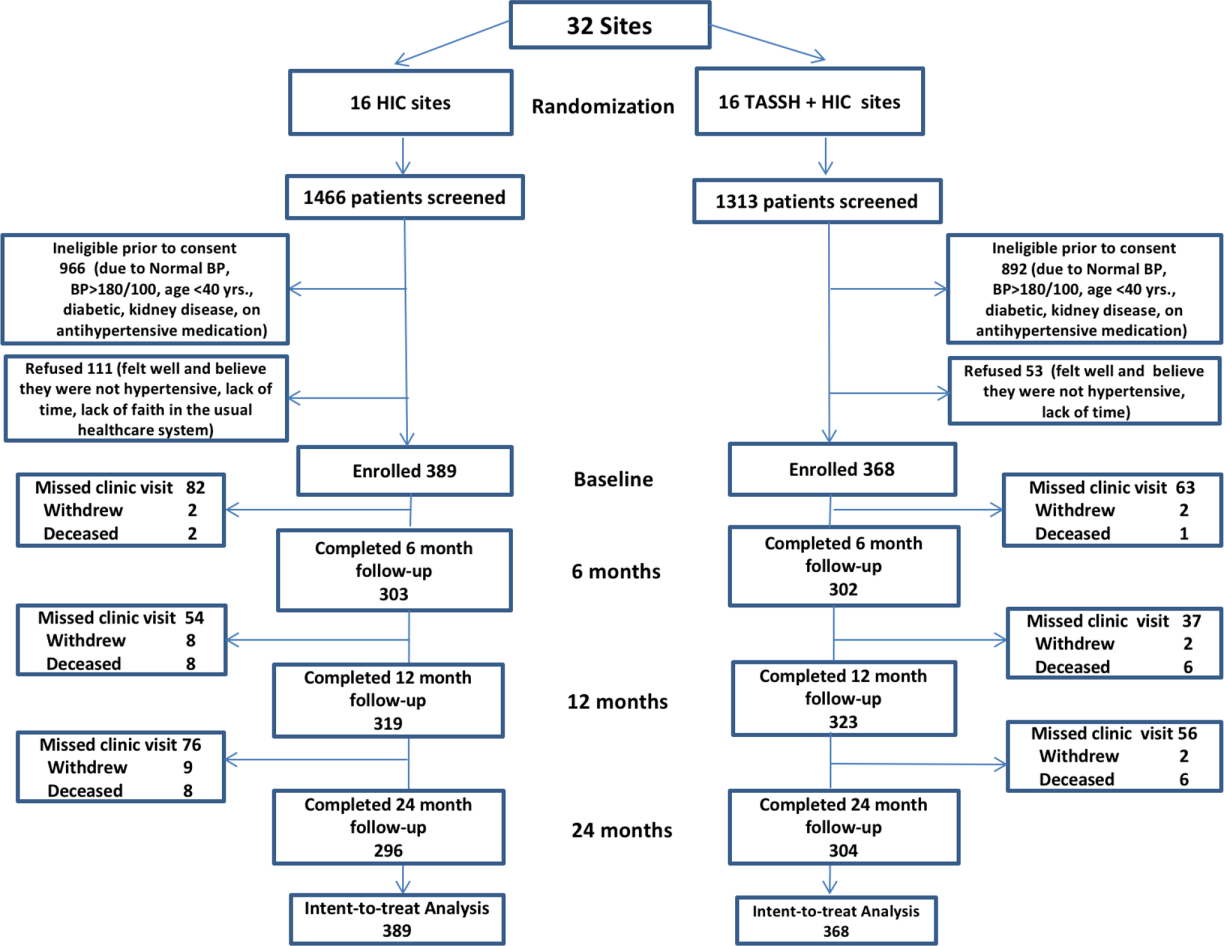
Consort diagram for patient flow (TASSH trial). BP, blood pressure; HIC, health insurance coverage; TASSH, task shifting strategy for hypertension control.

This study is reported as per the CONSORT guidelines for cluster trials (see S1 CONSORT Checklist). The study was a 2-arm pragmatic cluster randomized trial in which 32 CHCs within the Ghana Health Service Kumasi Metro Health Directorate (in the Ashanti Region) were randomly assigned to either HIC alone or TASSH plus HIC. Patients were recruited from participating CHCs if they were age 40 years or older, had newly diagnosed uncomplicated stage 1 hypertension (blood pressure [BP] 140–179/90–99 mm Hg), and had not received prior treatment. Patients with prior history of diabetes, coronary artery disease, stroke, heart failure, or proteinuria were excluded. The lower age limit of age 40 years was used because the WHO CV risk prediction charts [11] are based on and validated in adults 40 years and older. Complete study details have been published elsewhere [10]. Fig 1 shows the CONSORT diagram of patient flow. The study was approved by the institutional review boards of New York University and Kwame Nkrumah University of Science and Technology in Ghana. All patients provided informed consent, and the trial data were monitored by an independent data safety monitoring board. Patient recruitment occurred between November 28, 2012, and June 11, 2014, and the last patient follow-up was completed October 7, 2016. This study is registered in ClinicalTrials.gov (NCT01802372).

### Randomization and masking

The 32 CHCs (clusters) were pair-wise matched with respect to rural or urban location, and the CHCs in each matched pair were randomly assigned in a 1:1 ratio to either HIC or TASSH + HIC. The 32 CHCs were randomized in waves (cohorts) of 8 CHCs every 6 months for 2 years. The randomization sequence was electronically generated by the study statistician, and kept in sealed opaque envelopes away from the study sites, following CONSORT guidelines. Given the nature of the intervention, neither participants nor investigators were masked to the group assignment.

### Description of the interventions

Full details of both interventions are provided elsewhere [10]. We describe each below.

#### HIC group

Patients in this group received health insurance coverage for 12 months via Ghana’s national health insurance scheme, which provided access to primary care, medical consultations, laboratory tests, and medications to the patients at low cost [12]. The health insurance coverage was supplemented by scheduled nurse visits (every 3 months), during which patients’ BP was taken by the nurses, and those with elevated BP were encouraged to follow up with their physicians. The patients also received education materials about hypertension.

#### TASSH + HIC group

Patients in this group also received health insurance coverage in addition to the WHO CVD Package that was delivered by trained nurses. The WHO CVD Package includes clinical decision support for management of CVD via easy-to-follow algorithms, lifestyle counseling, and drug treatment protocols delivered by trained community health nurses for 1 year [8]. First, the nurses inquired about the patients’ medical history of prior heart attack, angina, heart failure, stroke, or diabetes. The nurses then conducted physical examinations including anthropometrics and BP measurements, following which they estimated the patients’ CV risk (low, medium, or high) using validated WHO risk charts based on urine dip stick for protein, fasting glucose, and plasma cholesterol [11]. Finally, the nurses referred those with high CV risk score to the district hospital for further management, and initiated antihypertensive study treatment for those at low/medium risk according to their BP level using any one of the following drugs: bendrofluazide, a diuretic; amlodipine, a calcium channel blocker (CCB); or lisinopril, an angiotensin-converting enzyme (ACE) inhibitor. Those with stage 1 hypertension received 2.5 mg bendrofluazide or 5 mg amlodipine, while those with stage 2 hypertension received combination therapy with 2 antihypertensive medications at low dose using a diuretic plus ACE inhibitor, a CCB plus ACE inhibitor, a diuretic plus a beta blocker (BB), or a CCB plus a BB, following JNC-7 guidelines [13]. The nurses titrated the medications (usually an increase in dose or addition of a second medication) every month, until BP control was achieved. Clinic visits occurred every month for 12 months. A total of 32 nurses at the 16 CHCs randomized to the TASSH + HIC group were trained in the implementation of the WHO CVD Package, hypertension treatment protocol, and strategies for lifestyle counseling during a 3-day training session, which occurred yearly for the duration of the study [14].

### Outcomes

The primary outcome was the mean change in SBP from baseline to 12 months, and the secondary outcomes were the rate of BP control and lifestyle behaviors (levels of physical activity, percent change in weight) at 12 months and the sustainability of SBP reduction at 24 months (1 year after the end of the trial). BP control was defined as BP < 140/90 mm Hg, following JNC-7 guidelines [13]. BP was assessed by a trained study staff with 3 measurements using a valid automated BP monitor taken following American Heart Association guidelines [15]. The mean of the 3 BP readings was used as the primary outcome measure. In addition to sociodemographics, other study measures included self-reported medication adherence and physical activity (measured with the Global Physical Activity Questionnaire, developed by WHO for use in LMICs) [16]. Patients completed baseline and 6-, 12-, and 24-month study visits, during which trained study staff assessed study outcomes.

### Statistical analysis

All analyses were conducted as planned and prespecified in the published study protocol [10] (S1 Text). We did not make any data-driven changes to analysis throughout the study period. Assuming that the TASSH + HIC group would have a 5 mm Hg greater reduction in SBP than the HIC group, an SBP standard deviation of 15 mm Hg [9], and an intra-class correlation (ICC) of 0.06, consistent with data from the WHO study [9], a sample size of 32 CHCs was estimated (with 20 patients per CHC, for a total of 640 patients) to satisfy power = 0.80, for the primary outcome. We fit a linear mixed effects regression model to the observed SBP data to predict the mean reduction in SBP between the 2 groups. All data were entered into a Microsoft Access 2010 database, and analyzed using IBM SPSS Statistics 20 and SAS Version 9 (PROC MIXED).

For the primary analysis, we tested the hypothesis that patients in the TASSH + HIC group would have greater SBP reduction than those in the HIC group at 12 months, using a linear mixed effects regression model with an unstructured covariance matrix error structure across 3 time points: baseline, 6 months, and 12 months. This analysis had 1 within-person factor, time (baseline and 6-month and 12-month follow-up); 1 between-group factor, treatment group; and the time by treatment interaction term. Additionally, patients were nested within CHCs, thus creating a 3-level model (observations nested within patients nested within CHCs). The nesting was handled by specifying an additional random intercept model across the 32 CHCs. Multilevel modeling software (e.g., SAS Version 9, PROC MIXED) was used to compute full-information maximum likelihood (FIML) estimates of the model parameters. To describe the pattern of results and to ensure that our modeling did not misrepresent the observed data, we compared mean BP data at baseline, 6 months, and 12 months using the observed data for both groups; we report these means and their 95% CIs.

In response to peer-review request, we conducted a sensitivity analysis in order to check the robustness of the primary results using regression-based single imputation in 2 different scenarios. For the first scenario, the missing data conformed to the pattern of the observed data, and the parameter estimates were based on complete data (observed and imputed) for all participants. For the second scenario, which we regard as the most conservative one, the imputed data for the HIC group were based on the observed patterns of change, whereas the imputed data for the TASSH + HIC group were constrained to have the same mean as baseline. That is, participants with missing BP data in the TASSH + HIC group were assumed to have shown no overall change in BP across 12 months. We imputed the missing BP data from all available BP data at the 3 time points (baseline, 6 months, and 12 months).

For the secondary analyses, we tested the hypothesis that the TASSH + HIC group would have greater BP control than the HIC group at 12 months, with a multilevel logistic regression that adjusted for clustering. For patients with missing data, we estimated BP control rate using multiply imputed data. To test the hypothesis that the SBP reduction from baseline to 12 months would be sustained at 24 months, we compared the difference between the 12- and 24-month SBP data for both groups. Finally, we tested the hypothesis that the TASSH + HIC group would report higher levels of physical activity and greater weight loss than the HIC group at 12 months, by fitting separate multilevel linear mixed models (observations within person within CHC) with 1 within-patient factor (time) and 1 between-patient factor (group) for the summary measures of physical activity and weight. All results were expressed with their 95% confidence intervals.

Data were deposited in the Dryad repository: https://doi.org/10.5061/dryad.16c9m51 [17].

### Trial registration

The trial was registered on February 17, 2013, and patient recruitment started in November 2012 and was completed in June 2014. Preliminary data analysis started in August 2016, about 2 years after completion of patient recruitment. Thus, trial registration occurred during the recruitment phase, prior to any analyses. The late registration was due to an error of omission. We hereby state that all future trials will be registered prospectively.

## Results

As shown in Fig 1, between November 28, 2012, and June 11, 2014, a total of 2,779 patients were screened and 757 enrolled, with an 85% completion rate at 12 months (88% for the TASSH + HIC group and 82% for the HIC group). Patients were followed up until October 7, 2016. The majority of the patients were middle-aged, were women, had elementary or high school education, and were employed. Mean BP, level of physical activity, and BMI were similar for both groups at baseline (see Table 1); the ICCs for systolic and diastolic BP were 0.04 and 0.07, respectively. A total of 64 nurses (2 per CHC) were trained to deliver the interventions [13]. Table 2 provides baseline characteristics for the CHC clusters.

**Table 1:** Baseline patient characteristics by treatment group.

Characteristic	Total (*N =* 757)	TASSH + HIC (*n =* 368)	HIC (*n =* 389)
Age (*n*)	755	368	387
Mean (SD)	58.03 (12.37)	59.20 (12.48)	56.92 (12.18)
Gender (*n*)	757	368	389
Female	60.2%	61.9%	58.6%
Body mass index (*n*)	412	226	186
Underweight (<18.5 kg/m^2^)	10.7%	12.8%	8.1%
Normal (18.5 to 24.9 kg/m^2^)	51.9%	52.7%	51.1%
Overweight (25.0 to 29.9 kg/m^2^)	26.2%	26.5%	25.8%
Obese (≥30 kg/m^2^)	11.2%	8.0%	15.0%
Marital status (*n*)	637	286	351
Separated/divorced/widowed	34.9%	41.3%	29.6%
Married/living as married	63.6%	57.3%	68.7%
Never married	1.6%	1.4%	1.7%
Education level (*n*)	695	324	371
No schooling	33.5%	35.2%	32.1%
Elementary/high school	60.3%	60.2%	60.4%
Post-high school	6.2%	4.6%	7.5%
Employment status (*n*)	704	336	368
Employed	66.8%	67.0%	66.6%
Household income per month (*n*)	152	92	60
<GH₵400 (<US$90)	70.4%	70.7%	70.0%
≥GH₵400 (≥US$90)	29.6%	29.3%	30.0%
Literacy (*n*)	747	363	384
Illiterate	64.9%	64.7%	65.1%
Smoking (*n*)	724	349	375
Current cigarette smokers	3.5%	2.6%	4.3%
Physical activity per week (*n*)	757	368	389
Active (≥600 MET min/week per WHO)	79.9%	78.8%	81.0%
Not active (<600 MET min/week per WHO)	20.1%	21.2%	19.0%
Blood pressure, mm Hg (*n*)	756	368	388
Systolic, mean (SD)	155.9 (12.1)	156.9 (11.8)	154.9 (12.3)
Diastolic, mean (SD)	89.6 (10.8)	89.0 (11.4)	90.4 (10.2)
Cardiovascular risk estimate (*n*)	617	287	330
<10%	79.4%	76.7%	81.8%
10% to 20%	11.0%	12.9%	9.4%
≥20% to 30%	7.9%	8.7%	7.3%
≥30% to 40%	1.3%	1.4%	1.2%
>40%	0.3%	0.3%	0.3%

BP, blood pressure; HIC, health insurance coverage; MET, metabolic equivalent; TASSH, task shifting strategy for hypertension control.

**Table 2:** Baseline characteristics of clinic clusters by treatment group.

Characteristic	Total	TASSH + HIC	HIC
**All clusters (*N*)**	**32**	**16**	**16**
Rural (%)	50%	50%	50%
Urban (%)	50%	50%	50%
Number of doctors on staff, mean (SD)	1.88 (2.12)	1.69 (1.85)	2.06 (2.41)
Number of nurses on staff, mean (SD)	75.66 (53.92)	75.25 (60.22)	76.06 (53.92)
Number of patients seen annually, mean (SD)	49,404.00 (50,262.49)	36,428.69 (27,883.54)	62,379.31 (63,909.31)
**Cohort 1 (*n*)**	**8**	**4**	**4**
Number of doctors on staff, mean (SD)	2.50 (2.56)	2.75 (2.63)	2.25 (2.87)
Number of nurses on staff, mean (SD)	81.63 (66.26)	99.00 (75.12)	64.25 (61.61)
Number of patients seen annually, mean (SD)	38,800.50 (24,448.85)	41,247.00 (26,194.57)	36,354.00 (26,317.76)
**Cohort 2 (*n*)**	**8**	**4**	**4**
Number of doctors on staff, mean (SD)	1.75 (1.98)	1.50 (1.92)	2.00 (2.31)
Number of nurses on staff, mean (SD)	75.50 (58.70)	69.00 (66.80)	82.00 (58.73)
Number of patients seen annually, mean (SD)	90,826.13 (85,060.43)	43,110.00 (49,059.08)	138,542.25 (91,673.20)
**Cohort 3 (*n*)**	**8**	**4**	**4**
Number of doctors on staff, mean (SD)	2.25 (2.55)	1.75 (1.71)	2.75 (3.40)
Number of nurses on staff, mean (SD)	68.38 (50.29)	56.50 (36.47)	80.25 (64.76)
Number of patients seen annually, mean (SD)	27,876.50 (11,261.49)	25,059.00 (7,392.28)	30,694.00 (14,835.81)
**Cohort 4 (*n*)**	**8**	**4**	**4**
Number of doctors on staff, mean (SD)	1.00 (1.195)	0.75 (0.957)	1.25 (1.50)
Number of nurses on staff, mean (SD)	77.13 (59.43)	76.50 (73.62)	77.75 (53.11)
Number of patients seen annually, mean (SD)	40,112.88 (22,592.89)	36,298.75 (21,840.48)	43,921.00 (25,985.07)

HIC, health insurance coverage; TASSH, task shifting strategy for hypertension control.

### Effect of TASSH + HIC versus HIC on SBP reduction at 12 months (primary outcome)

**Fig 2:**
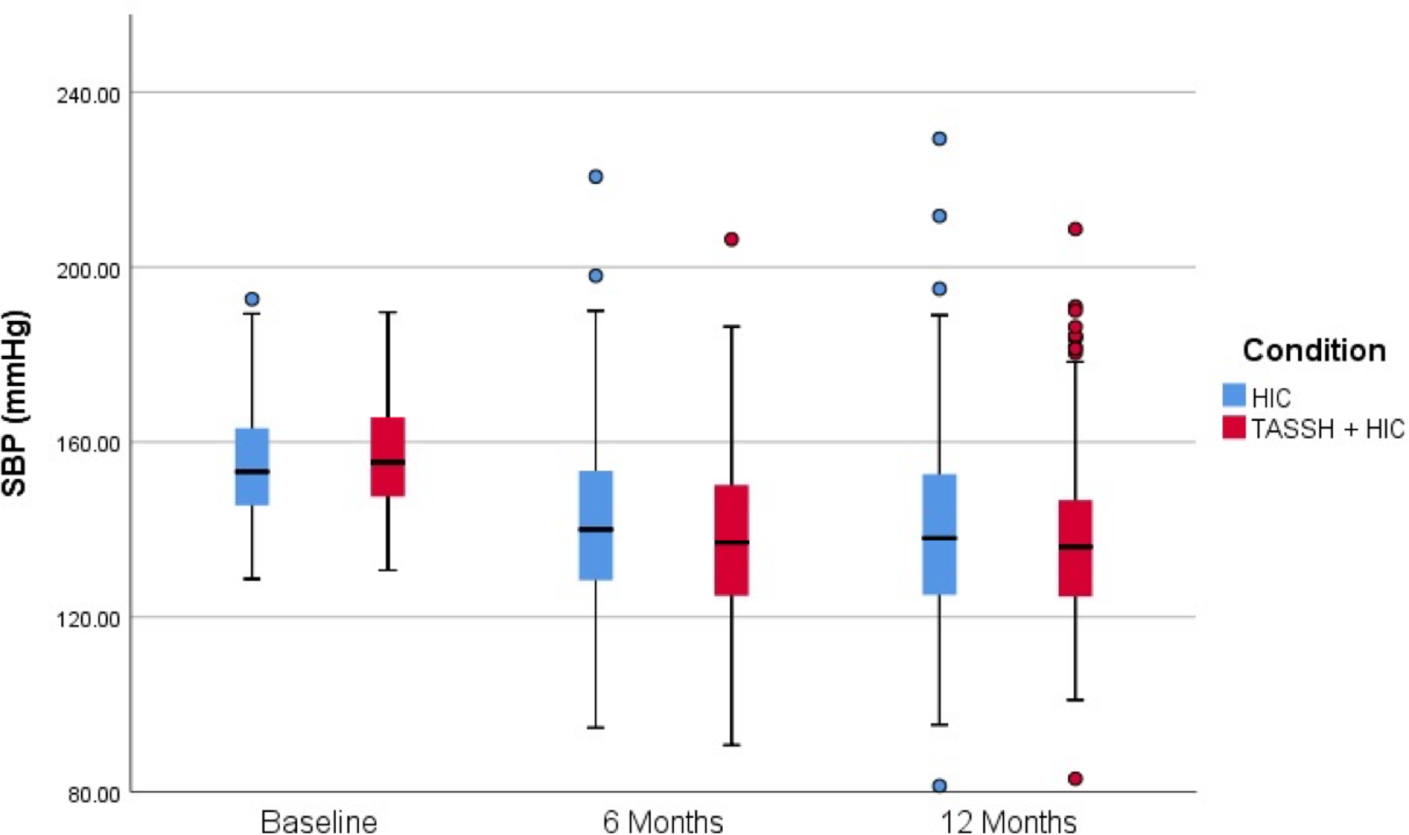
Change in SBP from baseline to 12 months. HIC, health insurance coverage; SBP, systolic blood pressure; TASSH, task shifting strategy for hypertension control.

Table 3 provides complete results of observed and estimated BP data (based on a linear model) for both groups at baseline, 6 months, and 12 months. Across both groups, there was a significant and sustained reduction in SBP from baseline to 12 months. As shown in Table 3, the estimated baseline SBP was 154.4 mm Hg for the HIC group versus 156.0 mm Hg for the TASSH + HIC group, a non-statistically significant difference (95% CI −0.69 to 3.92). At 12 months, the mean SBP was 135.6 mm Hg for the TASSH + HIC group and 137.6 mm Hg for the HIC group. We found a significant group by time interaction, with a greater SBP reduction in the TASSH + HIC group (−20.4 mm Hg; 95% CI −25.2 to −15.6) than in the HIC group (−16.8 mm Hg; 95% CI −19.2 to −15.6), and a significant between-group difference of −3.6 mm Hg (SE = 1.470*t*_(674)_ = −2.307; *p* = 0.021; 95% CI −6.1 to −0.5) (Table 3; Fig 2).

**Table 3:** Observed and adjusted mean systolic and diastolic blood pressure in the HIC and TASSH + HIC groups.

Measure and group	Mean (95% CI) blood pressure (mm Hg)			Mean (95% CI) difference from baseline		Effect size* (95% CI) (mm Hg)	
	Baseline	6 months	12 months	6 months	12 months	Baseline to 6 months	Baseline to 12 months
**Systolic blood pressure**							
TASSH + HIC	156.9 (155.7 to 158.1)	138.1 (135.1 to 141.0)	137.1 (134.1 to 140.1)	−18.6 (−21.2 to −15.9)	−19.5 (−21.9 to −17.2)	−4.3 (−8.2 to −0.3)	−2.9 (−6.9 to 1.0)
HIC	154.9 (153.7 to 156.2)	140.7 (137.8 to 143.7)	138.4 (135.4 to 141.4)	−14.3 (−17.4 to −11.1)	−16.6 (−19.9 to −13.3)	—	—
**Systolic blood pressure (estimated)**							
TASSH + HIC	156.0 (152.1 to 159.9)	145.8 (139.5 to 152.1)	135.6 (126.9 to 144.3)	−10.2 (−12.6 to −7.8)	−20.4 (−25.2 to −15.6)	−1.7 (−3.1 to −0.2)	−3.6 (−6.1 to −0.5)
HIC	154.4 (152.8 to 156.0)	146.0 (143.2 to 148.2)	137.6 (133.6 to 140.4)	−8.4 (−9.6 to −7.8)	−16.8 (−19.2 to −15.6)	—	—
**Diastolic blood pressure**							
TASSH + HIC	89.0 (87.8 to 90.2)	80.1 (77.7 to 82.5)	79.8 (77.3 to 82.3)	−9.1 (−11.0 to −7.1)	−9.3 (−10.5 to −8.0)	−2.3 (−5.1 to 0.6)	−0.6 (−2.9 to 1.7)
HIC	90.4 (89.4 to 91.4)	83.6 (81.2 to 85.9)	81.8 (79.3 to 84.3)	−6.8 (−9.0 to −4.6)	−8.7 (−10.7 to −6.6)	—	—
**Diastolic blood pressure (estimated)**							
TASSH + HIC	88.3 (84.0 to 91.1)	83.5 (77.4 to 86.9)	78.7 (70.8 to 82.7)	−4.8 (−6.6 to −4.2)	−9.6 (−13.2 to −8.4)	−0.6 (−1.2 to 0.6)	−1.2 (−2.4 to 1.2)
HIC	90.1 (88.3 to 91.8)	85.9 (82.9 to 88.2)	81.7 (77.5 to 84.6)	−4.2 (−5.4 to −3.6)	−8.4 (−10.8 to −7.2)	—	—

*N =* 757. TASSH + HIC sample size: baseline, *n* = 368; 6 months, *n* = 302; 12 months, *n* = 323. HIC sample size: baseline, *n* = 389; 6 months, *n* = 303; 12 months, *n* = 319.

*Effect size is defined as the net difference in systolic and diastolic blood pressure between the 2 groups at 6 and 12 months.

HIC, health insurance coverage; TASSH, task shifting strategy for hypertension control.

The sensitivity analyses conducted to check the robustness of the primary results also showed greater decrease in SBP across the 12 months for the TASSH + HIC group compared to the HIC alone group. Specifically, the parameter estimate indicated a greater reduction in SBP for TASSH + HIC compared to HIC alone (−0.27 mm Hg/month for the first scenario with a 95% CI of −0.48 to −0.06). Under the highly conservative second scenario, the parameter estimate indicated a smaller difference in SBP change between the TASSH + HIC and control group of −0.08 mm Hg/month (95% CI −0.30 to 0.13). Although the confidence interval in this highly conservative scenario contains 0, 70% of the values contained in the confidence interval are still consistent with a treatment effect.

### Comparison of SBP at 24 months between groups (secondary outcome)

Across all patients from both the HIC and TASSH + HIC groups, the mean change in SBP from the end of the trial at 12 months to the 24-month follow-up was −0.56 mm Hg, suggesting that the intervention effects in both groups were sustained 1 year after the end of the trial. For each group separately, the mean SBP at 24 months was 139.6 mm Hg for the TASSH + HIC group versus 137.9 mm Hg for the HIC group, a difference of 1.7 mm Hg (95% CI −1.36 to 4.82).

### Effect of TASSH + HIC versus HIC on BP control at 12 months (secondary outcome)

Both interventions led to significant improvement in the rate of BP control from baseline to 12 months: 52.4% with a 95% CI of 48.9% to 56.0%. The rate of BP control for the TASSH + HIC group was 55.2% (95% CI 50.0% to 60.3%) compared to 49.9% (95% CI 44.9% to 54.9%) for the HIC group, with a non-significant between-group difference of 5.2% (95% CI −1.8% to 12.4%).

### Effect of TASSH + HIC versus HIC on physical activity and weight loss at 12 months (secondary outcomes)

For these analyses, we fitted a doubly nested (time within patients within CHCs) linear mixed effects regression model to test if the change in level of physical activity from baseline to 12 months was different between groups. There was no evidence of differential change in level of physical activity between groups (difference in change in metabolic equivalent minutes/week = −0.04, 95% CI −0.12 to 0.05). Note that a metabolic equivalent of 1.0 is the amount of energy required to sit quietly, so this change is very small. For analyses of weight change from baseline to 12 months, we compared the difference in absolute weight change in kilograms between groups, controlling for baseline weight using a linear mixed model, which was adjusted for clustering of data within CHCs. The HIC group experienced a mean decrease in adjusted weight of −0.18 kg versus −0.06 kg for the TASSH + HIC group, yielding a non-significant difference of 0.12 (95% CI −0.80 to 1.06).

### Adverse events

A total of 21 adverse events (9 in the TASSH + HIC group and 12 in the HIC group) and 14 deaths (6 in the TASSH + HIC group and 8 in the HIC group) were reported (see Table 4). The known causes of death were stroke, kidney disease, bee sting, and alcohol overdose. Other than 2 patients in the TASSH + HIC group with persistent cough, a possible side effect of the ACE inhibitor lisinopril, the adverse events were not study-related.

**Table 4:** Adverse events for the TASSH trial.

Patient number	Group	Adverse event	Last visit number at the time of adverse event	Average BP reading at last visit (mm Hg)	Type	Intensity	Relationship to study	Resulted in medication change	Other comments regarding adverse event
1	HIC	Died	0	154/78	1	3	1	No	Unknown cause
2	TASSH + HIC	Persistent cough	2	138/82	2	2	2	Yes	None
3	TASSH + HIC	Persistent cough	1	159/95	2	2	2	Yes	None
4	HIC	Died	2	186/115	1	3	1	No	Unknown cause; died at home
5	TASSH + HIC	Died	1	149/80	1	3	1	No	Kidney Disease
6	HIC	Stroke	1	154/78	1	3	1	No	None
7	HIC	Died	4	122/72	1	3	1	No	Died en route to hospital
8	TASSH + HIC	Died	3	123/66	1	3	1	No	Unknown cause
9	HIC	Stroke	4	212/123	1	3	1	No	None
10	TASSH + HIC	Cancer	0	157/88	1	3	1	No	None
11	HIC	Died	3	165/105	1	3	1	No	Unknown cause; died at home
12	TASSH + HIC	Died	4	182/84	1	3	1	No	Unknown cause; died at home
13	TASSH + HIC	Died	3	140/80	1	3	1	No	Bee sting (attack)
14	HIC	Prostate enlargement	4	127/72	1	3	1	No	Hospitalized for prostate enlargement
15	HIC	Surgery	4	201/102	1	3	1	No	Hernia
16	HIC	Died	4	126/86	1	3	1	No	Unknown cause
17	HIC	Died	1	151/91	1	3	1	No	Stroke
18	HIC	Died	4	154/80	1	3	1	No	Stroke
19	TASSH + HIC	Died	4	147/74	1	3	1	No	Alcohol overdose
20	TASSH + HIC	Died	3	101/40	1	3	1	No	Unknown cause
21	HIC	Died	3	177/110	1	3	1	No	Unknown cause, but prior stroke

Last visit number at the time of adverse event or before it occurred: 00 = screening, 0 = baseline, 1 = 3 months, 2 = 6 months, 3 = 9 months, 4 = 12 months, 5 = 24 months, 6 = other (specify). Type: 1 = serious, 2 = non-serious. Intensity: 1 = mild, 2 = moderate, 3 = severe. Relationship to study: 1 = none, 2 = possible, 3 = probable, 4 = definite. Other than the 2 patients in the TASSH + HIC group with persistent cough—a possible side effect of the ACE inhibitor lisinopril—the adverse events were not study-related.

BP, blood pressure; HIC, health insurance coverage; TASSH, task shifting strategy for hypertension control.

## Discussion

In this pragmatic cluster randomized controlled trial, we evaluated the comparative effectiveness of 2 systems-level strategies for hypertension control among patients with newly diagnosed uncomplicated hypertension in 32 CHCs in Ghana. While both strategies led to significant reduction in SBP at 12 months, the TASSH + HIC group had a greater SBP reduction (−20.4 mm Hg; 95% CI −25.2 to −15.6) than the HIC group (−16.8 mm Hg; 95% CI −19.2 to −15.6), with a between-group difference of −3.6 mm Hg (95% CI −6.1 to −0.5). The SBP reduction for both groups was sustained at 24 months (1 year after completion of the trial), although there was no significant difference in SBP between the 2 groups at 24 months. The rate of BP control was similar for both groups (55.2% in the TASSH + HIC group versus 49.9% in the HIC group, *p =* 0.29), and there was no difference in lifestyle behaviors at 12 months.

Despite the growing burden of hypertension in SSA, implementation of task shifting strategies for hypertension control is lacking [18], other than the WHO study that evaluated the clinical effectiveness of the WHO CVD Package for hypertension control in 40 primary care practices in Nigeria and China [9]. To our knowledge, our study is the first pragmatic cluster randomized trial to evaluate the implementation of the WHO CVD Package by nurses in addition to provision of health insurance coverage within an established healthcare system in SSA. Our study also demonstrated, to our knowledge for the first time in SSA, the positive effect of health insurance coverage (plus regular nurse visits) on SBP reduction and BP control in a poor-resource setting. Although the WHO study and ours bear some similarities, our study is qualitatively different from the WHO study in several important ways. First, the duration of the WHO study was only 12 months, whereas our study demonstrated the sustainability of the SBP reduction 1 year after completion of the trial, at 24 months. Second, unlike the WHO study, which allowed initiation of drug therapy with only 1 low-dose diuretic, nurses in our study could initiate drug therapy with any of 3 drug classes (diuretic, ACE inhibitor, or calcium channel blocker). Third, unlike the WHO study, patients in our study received health insurance coverage to cover clinic visits and laboratory tests as needed, thus mitigating the negative effect of this important socioeconomic barrier to hypertension control [3].

Several factors may explain our findings. First, the roles of patient-level barriers (high out-of-pocket costs to pay for office visits, laboratory tests, and medications) [19] and systems-level barriers (poor access to care and shortage of healthcare providers) to hypertension control in SSA are well documented [19,20]. The provision of health insurance coverage to all patients in our study significantly mitigated the negative effects of these systems-level barriers. Second, poor patient knowledge and low rates of awareness of hypertension diagnosis are also well-documented barriers to hypertension control in SSA [3,19,21]. In a recent population-based survey of 2,400 people in Ghana, over 60% lacked knowledge of hypertension risk factors, and only 46% were aware of their hypertension diagnosis [22]. The implementation of regular clinic visits with nurses (every 3 months in the HIC group and monthly in the TASSH + HIC group) may have raised patients’ awareness and knowledge of their hypertension diagnosis, thus resulting in subsequent adoption of self-management behaviors and adherence to recommended treatment [23]. Finally, the greater reduction in SBP noted in the TASSH + HIC group versus the HIC group may be explained by medication intensification and the ability of nurses to titrate patients’ medications.

The strengths of our study are severalfold. First, the findings support an evidence-based systems-level strategy for hypertension control in the setting of acute shortage of healthcare workers in low-resource countries. We effectively integrated a task shifting strategy for hypertension control into Ghana’s healthcare system, with use of nurses who were employed within participating CHCs. This is an important demonstration of the ability of nurses to provide care for patients with uncomplicated hypertension. This strategy is potentially generalizable across Ghana because all nurses in CHCs in Ghana are employed by the Ghana Health Service. Second, the study design was a cluster randomized controlled trial with pragmatic outcomes of BP reduction and control, making replication possible. Third, the intervention was largely implemented by trained community health nurses, and thus has the potential for generalizability to other low-resource countries, particularly using a train-the-trainer model [20,24].

We should note the following limitations. First, the current lack of policy within Ghana Health Service to grant nurses prescribing power for antihypertensive medications for the management of patients with uncomplicated hypertension is a major challenge to scaling up this strategy across Ghana. A similar approach was successfully adopted for management of HIV in LMICs [6], and there is considerable evidence suggesting that task shifting of prescribing duties from doctors to trained nurses for initiation and maintenance of antiretroviral therapy was equally effective compared to care received from physicians [25]. It is imperative for countries in SSA to strongly consider this policy for management of hypertension, if we are to stave off its growing burden in the region. Second, we adopted a comparative effectiveness research approach to evaluate 2 active systems-level strategies for hypertension control without a usual care arm because we could not ethically justify withholding existing national health insurance coverage from eligible patients. This is an important limitation of the study because we could not ascertain with certainty that the SBP reduction noted in the group that received health insurance coverage alone was purely due to the access to care. Third, we did not conduct a cost-effectiveness analysis to determine the relative costs and benefits of each intervention. Future scale-up and widespread dissemination of these systems-level strategies will require a detailed cost–benefit analysis as a crucial step needed to ascertain the sustainability and scalability of both strategies over time. Other weaknesses include absence of information on the role of treatment intensification or medication adherence as potential mechanisms for the greater SBP reduction noted in the TASSH + HIC group. Because of the pragmatic nature of the study, we collected data on prescribed antihypertensive medications only among patients in the TASSH + HIC group. Similarly, as part of the intervention we provided free medications for patients in the TASSH + HIC group for only 12 months, and did not collect data on the number of patients who discontinued their medications because of cost after the end of the trial. That said, the majority of patients (82%) who received free BP medications provided 24-month follow-up data (1 year after discontinuation of provision of free BP medications), and among these, there were no cases of medication side effects or adverse effects related to drug withdrawal syndrome. Finally, we could not ascertain the role of dietary changes as potential mechanisms because the data were largely uninterpretable. Specifically, the questionnaire we utilized for data collection on diet does not account for the type of diet that is predominant in rural and sub-urban areas of Ghana.

Findings from our study have significant public health and policy implications for management of hypertension in SSA. Most importantly, despite the beneficial effect of BP reduction on CV morbidity and mortality [26], hypertension control rates remain unacceptably low across the region [21,23,27]. For example, a review of population-based studies in Ghana reported abysmally low BP control rates, between 1.7% and 12.7% [28], which are much lower than the 50% BP control rate achieved in our study. Furthermore, patients in both arms of our study experienced a SBP reduction of at least 10 mm Hg that was sustained over 24 months. According to a meta-analysis of 147 randomized trials of use of antihypertensive medications for CVD prevention, this magnitude of SBP reduction is associated with a 41% and 22% reduction in stroke and ischemic heart attacks, respectively [29]. Thus, the public health benefit of implementing the WHO CVD Package plus providing health insurance across CHCs in Ghana is potentially significant. Currently, however, most countries in SSA do not have national health insurance coverage, and where such exists, the per capita investment in healthcare is quite low [30]. Incorporating delivery of the WHO CVD Package as part of the duties of nurses within existing healthcare systems in SSA represents a viable implementation strategy, particularly in countries like Tanzania, Kenya, Ethiopia, Cameroon, and Nigeria, where task shifting of primary care duties to non-physician health workers already exists [31]. Moreover, the reliability of this strategy has been previously established by WHO [20], thus making its generalizability and scale-up possible.

In conclusion, we demonstrated that 2 evidence-based systems-level strategies, provision of health insurance coverage with and without a nurse-led task shifting strategy for hypertension control, led to significant reduction in SBP and improvement in BP control among patients with uncontrolled hypertension in Ghana. The addition of a nurse-led task shifting strategy was associated with a greater reduction in SBP than the provision of health insurance coverage alone. Future research should address the cost-effectiveness of these strategies and their potential scale-up across Ghana and other countries in SSA.

## Supporting information

S1 CONSORT Checklist (DOCX)

S1 Text Prespecified analysis plan. (DOCX)

## Abbreviations

ACE: angiotensin-converting enzyme
BP: blood pressure
CHC: community health center
CV: cardiovascular
CVD: cardiovascular disease
HIC: health insurance coverage
ICC: intra-class correlation
LMICs: low- and middle-income countries
SBP: systolic blood pressure
SSA: sub-Saharan Africa
TASSH: task shifting strategy for hypertension control
WHO: World Health Organization
WHO CVD Package: WHO CVD-Risk Management Package

## References

[1] GBD 2015 Disease and Injury Incidence and Prevalence Collaborators. Global, regional, and national incidence, prevalence, and years lived with disability for 310 diseases and injuries, 1990–2015: a systematic analysis for the Global Burden of Disease Study 2015. Lancet. 2016;388(10053):1545–602. doi: 10.1016/S0140-6736(16)31678-6 27733282 10.1016/S0140-6736(16)31678-6 PMC5055577

[2] Anyangwe SC, Mtonga C. Inequities in the global health workforce: the greatest impediment to health in sub-Saharan Africa. Int J Environ Res Public Health. 2007;4(2):93–100. 17617671 10.3390/ijerph2007040002 PMC3728573

[3] Mendis S, Abegunde D, Oladapo O, Celletti F, Nordet P. Barriers to management of cardiovascular risk in a low-resource setting using hypertension as an entry point. J Hypertens. 2004;22(1):59–64. 15106795 10.1097/00004872-200401000-00013

[4] World Health Organization. World health statistics 2015. Geneva: World Health Organization; 2015 [cited 2016 Sep 12]. Available from: http://www.who.int/gho/publications/world_health_statistics/2015/en/.

[5] World Health Organization. Task shifting: global recommendations and guidelines. Geneva: World Health Organization; 2008 [cited 2017 Jan 18]. Available from: http://www.who.int/healthsystems/TTR-TaskShifting.pdf.

[6] Callaghan M, Ford N, Schneider H. A systematic review of task-shifting for HIV treatment and care in Africa. Hum Resour Health. 2010;8:8 doi: 10.1186/1478-4491-8-8 20356363 10.1186/1478-4491-8-8 PMC2873343

[7] Lekoubou A, Awah P, Fezeu L, Sobngwi E, Kengne AP. Hypertension, diabetes mellitus and task shifting in their management in sub-Saharan Africa. Int J Environ Res Public Health. 2010;7(2):353–63. doi: 10.3390/ijerph7020353 20616978 10.3390/ijerph7020353 PMC2872286

[8] World Health Organization. WHO CVD risk management package for low- and medium-resource settings Geneva: World Health Organization; 2002 [cited 2016 Apr 15]. Available from: http://whqlibdoc.who.int/publications/2002/9241545852.pdf?ua=1.

[9] Mendis S, Johnston SC, Fan W, Oladapo O, Cameron A, Faramawi MF. Cardiovascular risk management and its impact on hypertension control in primary care in low-resource settings: a cluster-randomized trial. Bull World Health Organ. 2010;88(6):412–9. doi: 10.2471/BLT.08.062364 20539854 10.2471/BLT.08.062364 PMC2878142

[10] Ogedegbe G, Plange-Rhule J, Gyamfi J, Chaplin W, Ntim M, Apusiga K, et al A cluster-randomized trial of task shifting and blood pressure control in Ghana: study protocol. Implement Sci. 2014;9:73 doi: 10.1186/1748-5908-9-73 24923300 10.1186/1748-5908-9-73 PMC4063247

[11] World Health Organization. WHO/ISH risk prediction charts for 14 WHO epidemiological sub-regions Geneva: World Health Organization; 2007 [cited 2018 Mar 28]. Available from: http://ish-world.com/downloads/activities/colour_charts_24_Aug_07.pdf.

[12] Nguyen HT, Rajkotia Y, Wang H. The financial protection effect of Ghana National Health Insurance Scheme: evidence from a study in two rural districts. Int J Equity Health. 2011;10:4 doi: 10.1186/1475-9276-10-4 21247436 10.1186/1475-9276-10-4 PMC3031235

[13] Chobanian AV, Bakris GL, Black HR, Cushman WC, Green LA, Izzo JL Jr, et al Seventh report of the Joint National Committee on Prevention, Detection, Evaluation, and Treatment of High Blood Pressure. Hypertension. 2003;42(6):1206–52. doi: 10.1161/01.HYP.0000107251.49515.c2 14656957 10.1161/01.HYP.0000107251.49515.c2

[14] Gyamfi J, Plange-Rhule J, Iwelunmor J, Lee D, Blackstone SR, Mitchell A, et al Training nurses in task-shifting strategies for the management and control of hypertension in Ghana: a mixed-methods study. BMC Health Serv Res. 2017;17(1):104 doi: 10.1186/s12913-017-2026-5 28148255 10.1186/s12913-017-2026-5 PMC5288999

[15] Pickering TG, Hall JE, Appel LJ, Falkner BE, Graves J, Hill MN, et al Recommendations for blood pressure measurement in humans and experimental animals: Part 1: blood pressure measurement in humans: a statement for professionals from the Subcommittee of Professional and Public Education of the American Heart Association Council on High Blood Pressure Research. Hypertension. 2005;45(1):142–61. doi: 10.1161/01.HYP.0000150859.47929.8e 15611362 10.1161/01.HYP.0000150859.47929.8e

[16] World Health Organization. Global Physical Activity Questionnaire (GPAQ). Geneva: World Health Organization; 2018 [cited 2018 Mar 28]. Available from: http://www.who.int/chp/steps/GPAQ_EN.pdf.

[17] Ogedegbe GO, Plange-Rhule J, Gyamfi J, Chaplin W, Ntim M, Apusiga K, et al Health insurance coverage with or without a nurse-led task shifting strategy for hypertension control: a pragmatic cluster randomized trial in Ghana [dataset] 2018 [cited 2018 Mar 28]. Dryad Digital Repository. Available from: https://doi.org/10.5061/dryad.16c9m51. 10.1371/journal.pmed.1002561 PMC5929500 29715303

[18] Ogedegbe G, Gyamfi J, Plange-Rhule J, Surkis A, Rosenthal DM, Airhihenbuwa C, et al Task shifting interventions for cardiovascular risk reduction in low-income and middle-income countries: a systematic review of randomised controlled trials. BMJ Open. 2014;4(10):e005983 doi: 10.1136/bmjopen-2014-005983 25324324 10.1136/bmjopen-2014-005983 PMC4202019

[19] Iwelunmor J, Plange-Rhule J, Airhihenbuwa CO, Ezepue C, Ogedegbe O. A narrative synthesis of the health systems factors influencing optimal hypertension control in sub-Saharan Africa. PLoS ONE. 2015;10(7):e0130193 doi: 10.1371/journal.pone.0130193 26176223 10.1371/journal.pone.0130193 PMC4503432

[20] Abegunde DO, Shengelia B, Luyten A, Cameron A, Celletti F, Nishtar S, et al Can non-physician health-care workers assess and manage cardiovascular risk in primary care? Bull World Health Organ. 2007;85(6):432–40. doi: 10.2471/BLT.06.032177 17639240 10.2471/BLT.06.032177 PMC2636344

[21] Kayima J, Wanyenze RK, Katamba A, Leontsini E, Nuwaha F. Hypertension awareness, treatment and control in Africa: a systematic review. BMC Cardiovasc Disord. 2013;13:54 doi: 10.1186/1471-2261-13-54 23915151 10.1186/1471-2261-13-54 PMC3750220

[22] Lamptey P, Laar A, Adler AJ, Dirks R, Caldwell A, Prieto-Merino D, et al Evaluation of a community-based hypertension improvement program (ComHIP) in Ghana: data from a baseline survey. BMC Public Health. 2017;17(1):368 doi: 10.1186/s12889-017-4260-5 28454523 10.1186/s12889-017-4260-5 PMC5410035

[23] Iwelunmor J, Blackstone S, Gyamfi J, Airhihenbuwa C, Plange-Rhule J, Tayo B, et al A concept mapping study of physicians’ perceptions of factors influencing management and control of hypertension in sub-Saharan Africa. Int J Hypertens. 2015;2015:412804 doi: 10.1155/2015/412804 26550488 10.1155/2015/412804 PMC4621343

[24] Green LW. Making research relevant: if it is an evidence-based practice, where’s the practice-based evidence? Fam Pract. 2008;25(Suppl 1):i20–4. 18794201 10.1093/fampra/cmn055

[25] Kredo T, Adeniyi FB, Bateganya M, Pienaar ED. Task shifting from doctors to non-doctors for initiation and maintenance of antiretroviral therapy. Cochrane Database Syst Rev. 2014;7:CD007331. 10.1002/14651858.CD007331.pub3 PMC11214583 24980859

[26] Blood Pressure Lowering Treatment Trialists’ Collaboration. Blood pressure-lowering treatment based on cardiovascular risk: a meta-analysis of individual patient data. Lancet. 2014;384(9943):591–8. doi: 10.1016/S0140-6736(14)61212-5 25131978 10.1016/S0140-6736(14)61212-5

[27] Kearney PM, Whelton M, Reynolds K, Muntner P, Whelton PK, He J. Global burden of hypertension: analysis of worldwide data. Lancet. 2005;365(9455):217–23. doi: 10.1016/S0140-6736(05)17741-1 15652604 10.1016/S0140-6736(05)17741-1

[28] Addo J, Agyemang C, Smeeth L, de-Graft Aikins A, Edusei AK, Ogedegbe O. A review of population-based studies on hypertension in Ghana. Ghana Med J. 2012;46(2 Suppl):4–11. 23661811 PMC3645150

[29] Law M, Morris J, Wald N. Use of blood pressure lowering drugs in the prevention of cardiovascular disease: meta-analysis of 147 randomised trials in the context of expectations from prospective epidemiological studies. BMJ. 2009;338:b1665 doi: 10.1136/bmj.b1665 19454737 10.1136/bmj.b1665 PMC2684577

[30] World Health Organization. State of health financing in the African region Geneva: World Health Organization; 2013 [cited 2017 Mar 23]. Available from: http://apps.who.int/iris/bitstream/10665/101282/1/9789290232131.pdf.

[31] Carapinha JL, Ross-Degnan D, Desta T, Wagner AK. Health insurance systems in five sub-Saharan African countries: medicine benefits and data for decision making. Health Policy. 2011;99(3):193–202. doi: 10.1016/j.healthpol.2010.11.009 21167619 10.1016/j.healthpol.2010.11.009

