# Supplementary material for: Health insurance coverage with or without a nurse-led task shifting strategy for hypertension control: A pragmatic cluster randomized trial in Ghana
Source: PLoS Med. 2018 May 1;15(5):e1002561. doi: 10.1371/journal.pmed.1002561 (PMC5929500; doi:10.1371/journal.pmed.1002561)
Supplement: S1 Text — (DOCX) [file pmed.1002561.s002.docx]

**S1 Text: Pre-Specified Analysis Plan**

**Outcome Measures**

The primary outcome is the mean change in systolic BP from baseline to 12 months. The

secondary outcomes include proportion of patients with adequate BP control at 12 months; levels

of physical activity, percent change in weight and dietary intake of fruits and vegetables at 12

months; and BP control rates at 24 months. BP control is defined as BP<140/90 mmHg

following JNC-7 guidelines. BP readings will be assessed with validated automated BP

device. All study outcomes will be assessed at baseline, 6 months, and 12 months.

**Sample size and power analysis**

The study is a 2-arm cluster randomized trial with the health center as the unit of

randomization. Thus, there are two important sample size estimates, N the number of patients

and K, the number of clinics, with the patients nested within the K clinics. The sample size

calculations is based on a two-tailed alpha level of p = .05, and power at .80. The effect size is

based on a greater reduction in SBP of 5 mm Hg in the IG compared to the CG. A standard

deviation of SBP of 15 mm Hg [10], which yields a standardized effect size of BP change of d =

.33 and an ICC of .06 consistent with the data from the WHO-sponsored Mendis et. al trial in

Nigeria [10] was used to estimate the sample size. Based on this information the combinations of

clinics and patients/clinic in Table 1 satisfies our desire for power = .80. Table 1 illustrates the

flexibility in sample size estimates with respect to both recruitment and attrition. For example, if

the recruitment goal is an average of 30 patients in each clinic, but only an average of 20 per site

was achieved, it means 6 more clinics (26 + 6 = 32) will need to be randomized to maintain a

statistical power of 0.80. Likewise if an attrition rate of 25% among the 20 patients was

experiences, then 4 more clinics (32 + 4 = 36) will need to be recruited in order to maintain the

same power level with an average of 15 completers /clinic.

**Statistical Methods**

Analysis for Primary Hypotheses: We hypothesize that patients in the intervention group will

have greater systolic BP reduction than those in the control group at 12 months: This analysis

will be accomplished with a multilevel MANOVA (unstructured covariance matrix across three

time points baseline, 6 and 12 months). This analysis will have one within-person factor – Time

(baseline, 6-month, and 12-month follow-up) and one primary between-patient factor

(Randomization Group). The outcome measures in this analysis will be systolic BP. Additionally

the patients will be nested within clinics creating a 3-level analytic model (observations nested

within patients nested within clinics). Multilevel modeling software (SAS, Version 9, PROC

MIXED) will be used to compute full-information maximum likelihood (FIML) estimates of the

model parameters . The PROC MIXED procedure will use an error structure that allows for

the possibility of group differences in (a) the error variances at follow-up; and (b) the serial

correlations of baseline BP with 6- and 12-month BP. The primary test concerns the Group X

Time interaction, and the resulting F-test will provide the primary “intent-to-treat” test of the

hypothesis. If this is statistically significant at the two-tailed α=.05 level, for ease of

interpretation, we will estimate and report the magnitude of the treatment effect, with 95% CI for

SBP. Ideally, the randomization of participants to treatment arm and the absence of significant

selection and/or attrition biases will obviate the need for any covariates in the analysis. However,

in the event that the above-described probit analyses indicate one or more sources of potential

bias, the predicted values of those analyses will be included as covariates in the MANOVA

(including their interactions with the within-person factors).

**Analysis for Secondary Hypotheses**

Secondary Hypothesis 1: Patients in the intervention group will have higher BP control rate than

those in the control group at 12 months: This hypothesis will be assessed with a 2 (group) x 2

(control) chi-square analyses as there is no time factor in these analyses. However, we will use

multilevel logistic regression to control for the nesting of patients within clinics. BP control will

be defined as occurring when systolic BP<140 mm Hg and diastolic BP<90 mm Hg at 12

months. Effect sizes will be reported as odds ratios with 95% CI. The covariates describe above

will be included in the analyses as needed. For those participants without BP data at 12 months,

we will estimate their BP control using data from previous time points.

Secondary Hypothesis 2: Patients in the intervention group will have higher levels of physical

activity, greater weight loss, and intake of fruits and vegetables than those in the control group

at 12 months: Similar to the primary hypothesis testing, we will perform separate multilevel

MANOVAs (observations within person within health center) with one within-patient factor

(Time) and one between-patient factor (Group) for a) the summary measure of physical activity,

b) weight, and c) the summary measure of intake of fruits and vegetables. For each analysis, we

will allow for group differences in the variance of the outcome at follow-up and the serial

correlation of baseline with follow-up assessments. Because changes in weight tend to be

proportional to baseline weight, we will follow the widely used strategy of analysing the percent

change in weight; thus, there will be no within-patient component to this analysis per se, but we

will allow for group differences in the SD of percent change. Similar to the analysis for the

primary hypothesis, if evidence of selection or attrition biases or group differences resulting from

randomization is detected, predicted values of the corresponding probit analysis will be

incorporated as covariates into these analyses. However, we may consider introducing an

adjustment to protect against the experiment-wide risk of a Type I error. If so, we would use

Holm’s modified Bonferroni procedure, which is less conservative than the traditional

Bonferroni procedure, but still controls for the experiment-wide α-level .

Secondary Hypothesis 3: Patients in the intervention group will maintain higher BP control

rates and greater reduction of both SBP and DBP than those in the control group at 24 months:

These analyses will be identical to those for the primary hypothesis except that an additional 24

month time point will be added to the multilevel model. We will consider a spline regression

model to assess the possibility that there will be no further linear decrease in BP in the groups,

but simply a maintenance of the changes achieved at 12 months.
